# Evaluating Racial Disparities in Implementation and Monitoring of a Remote Blood Pressure Program in a Pregnant Population—A Retrospective Cohort Study

**DOI:** 10.31486/toj.23.0111

**Published:** 2024

**Authors:** Elizabeth Howard, Veronica Gillispie-Bell, Susan Olet, Beth Glenn, Nariman Ammar, Eboni G. Price-Haywood

**Affiliations:** ^1^ Ochsner-Xavier Institute for Health Equity and Research, Ochsner Clinic Foundation, New Orleans, LA; ^2^ Department of Obstetrics and Gynecology, Ochsner Clinic Foundation, New Orleans, LA; ^3^ The University of Queensland Medical School, Ochsner Clinical School, New Orleans, LA

**Keywords:** *Blood pressure monitoring–ambulatory*, *hypertension–pregnancy induced*, *postpartum period*, *pregnancy*, *race factors*

## Abstract

**Background:** Whether remote blood pressure (BP) monitoring can decrease racial disparities in BP measurement during pregnancy and the postpartum period remains unclear. This study evaluated whether Black and White patients enrolled in the Connected Maternity Online Monitoring (CMOM) program showed improvements in BP ascertainment and interval.

**Methods:** A retrospective cohort of 3,976 pregnant patients enrolled in CMOM were compared to matched usual care patients between January 2016 and September 2022 using electronic health record data. The primary outcomes were BP ascertainment (number of BP measurements) and BP interval (time between BP measurements) during pregnancy and the postpartum period. The proportion of patients with a hypertensive disorder of pregnancy who checked their BP within 7 days of discharge following delivery was also assessed.

**Results:** Enrollment in CMOM was lower among Black patients than White patients (42.1% vs 54.7%, *P*<0.0001). Patients in the CMOM group had more BP measurements than patients in the usual care group during pregnancy (rate ratio=1.78, 95% CI 1.74-1.82) and the postpartum period (rate ratio=1.30, 95% CI 1.23-1.37), with significant improvements for both Black and White patients enrolled in CMOM compared to patients in usual care. The CMOM intervention did not result in an improvement in 7-day postpartum adherence to checking BP for Black patients (risk ratio=1.03, 95% CI 0.94-1.11) as it did for White patients (risk ratio=1.09, 95% CI 1.01-1.17).

**Conclusion:** Remote BP monitoring programs are a helpful tool to improve the frequency of BP measurements and shorten intervals between measurements during the prenatal and postpartum periods for all patients. Future evaluation is needed to determine the barriers to offering the program to and enrolling Black patients.

## INTRODUCTION

Hypertensive disorders of pregnancy are a leading cause of maternal morbidity and mortality, with the incidence in the United States increasing from 13.3% to 15.9% between 2017 and 2019.^1^ Black patients are at higher odds for preeclampsia,^2^ eclampsia,^3^ and severe maternal morbidity^2-6^ compared to their White peers and are at least 2.6 times more likely to die a pregnancy-related death.^7-13^ Disparities also exist during the postpartum period, with Black individuals at increased odds of postpartum readmission because of hypertension.^14,15^ Deaths caused by hypertensive disorders of pregnancy most commonly occur between days 0 to 6 postpartum.^16^

Access to maternity care is an important predictor of poor maternal outcomes. Women living in rural and deprived areas have 60% and 97% higher maternal mortality risks than women living in urban and affluent areas, respectively.^11^ Furthermore, socially and economically vulnerable patients who are younger, have a minority ethnic background, and depend on state-funded health insurance have higher rates of prenatal and postpartum visit nonattendance.^17,18^

Timely diagnosis and treatment of hypertension during pregnancy and continued monitoring in the postpartum period are key to improving outcomes. Strategies to improve monitoring must be developed through a lens of equity so disparities are not increased further. Home-based blood pressure (BP) monitoring—a feasible, valid, acceptable, and cost-effective way to diagnose and monitor BP during pregnancy—has been shown to reduce prenatal hospital admissions, induction of labor, and antenatal visits^19-22^ and to improve attendance at postpartum hypertension visits among non-Hispanic Black women.^23,24^ Whether remote monitoring technology can significantly decrease racial disparities in BP measurement is unclear.

This study evaluated whether Black and White patients who are enrolled in Connected Maternity Online Monitoring (CMOM), a remote monitoring program at a hospital system in Louisiana, USA, showed improvements in the frequency of BP measurement and the interval between measurements during pregnancy and the postpartum period.

## METHODS

This retrospective observational cohort study included adult patients with a singleton pregnancy and 1 pregnancy record who were eligible for enrollment in the remote BP monitoring program CMOM during the study period and delivered after 20 weeks’ gestation at a participating hospital between January 1, 2016, and September 5, 2022. Patients who did not have a complete 6-week postpartum period during the time frame were excluded. Six weeks following delivery was chosen to reflect the traditional comprehensive postpartum visit. The study was approved by an institutional review board.

Patients who were eligible for the program met the following criteria: prenatal visit with a provider whose primary clinic offered CMOM, active pregnancy episode in the Epic (Epic Systems Corporation) electronic health record (EHR), gestational age between 10 weeks and 20 weeks at enrollment, and possession of a Bluetooth-enabled personal smartphone (iOS or Android). Patients meeting the criteria were offered program enrollment by their prenatal care provider (obstetrician, nurse practitioner, or midwife).

Once enrolled, patients were instructed to download and set up 2 smartphone applications, iHealth MyVitals (iHealth Labs Inc) and MyChart (Epic Systems Corporation), sign an informed consent, and acquire and set up their CMOM kit that includes a Bluetooth-enabled BP cuff (iHealth Ease BP Cuff, iHealth Labs Inc) and scale (iHealth Nexus Scale, iHealth Labs Inc). A program guide that includes guidance on how to set up the BP cuff is included, and online support with a video on how to take an accurate BP reading is available. CMOM participants are expected to digitally record at least 1 BP reading per week. Participants receive text reminders if no data is received in 8 days. The data from the remote BP units transmit directly to the EHR. The patient's obstetric care team is alerted of validated high BP readings (≥140/90 mm Hg) through the staff Epic In Basket (Epic Systems Corporation). Patients are alerted to high BP readings with automated instructions via text to recheck their BP 10 to 15 minutes later. If their BP is still elevated, they receive another message instructing them to contact their physician's office. The program continues through delivery until the patient's 4- to 6-week postpartum visit.

The study cohort included pregnant patients enrolled in CMOM and patients receiving usual care who were eligible to participate in CMOM. Usual care patients received routine prenatal care through their obstetric provider, and all BP measurements were acquired in clinic. BP measurements for patients enrolled in CMOM included both remote and clinic measurements. All data for this study, including remote and office BP readings, demographics, comorbidities, maternal care, and maternal health outcomes, were collected from the EHR and retrieved through electronic query. A total of 11,081 eligible patients were identified after inclusion and exclusion criteria were applied. The 1:1 optimal matching approach was used to match all 3,976 patients in the CMOM group to 3,976 patients from the usual care group using propensity scores obtained by fitting a logistic regression adjusting for age, preexisting hypertension status at initial visit, and health insurance coverage type to address potential selection bias and confounding. These variables were chosen based on a priori clinical expertise and significant bivariate associations with both race and the outcomes. Preexisting hypertension status was defined as having preexisting hypertension complicating pregnancy, childbirth, and the puerperium (*International Classification of Diseases-10* diagnosis code O10) listed in the problem list or encounter diagnoses.

The primary outcomes were BP ascertainment and interval. BP ascertainment was defined as the average number of BP measurements during the prenatal and postpartum periods. BP interval was defined as the average time interval in days between BP measurements during the prenatal and postpartum periods. The outcomes during the postpartum period were limited to patients with a diagnosed hypertensive disorder of pregnancy during the current pregnancy to reflect the population requiring BP monitoring following delivery. Additionally, we assessed postpartum adherence according to the 2018 American College of Obstetrics and Gynecologists recommendation that BP be evaluated between 3 to 10 days postpartum for all women by evaluating the proportion of patients diagnosed with a hypertensive disorder of pregnancy who checked their BP within 7 days of discharge from the hospital.^25^ These process outcomes were chosen to reflect the CMOM program goal of frequent BP monitoring throughout pregnancy and the postpartum period to inform clinical decision-making by obstetrics providers.

Race and ethnicity were self-reported by patients during clinical assessments with patient-facing staff or directly through the patient portal. Patients select categories according to the US Census Bureau and were able to select multiple options. For the purposes of this analysis, only patients who identified their race as solely Black or White were included because of small numbers in the other race categories (n=942).

Patient characteristics are presented as mean ± SD or counts and proportions where appropriate. Comparisons were performed using the chi-square test or Fisher exact test for categorical variables and the Wilcoxon rank sum test for continuous variables. Continuous variables were assessed for normality using the Shapiro-Wilk and Kolmogorov-Smirnov tests. To examine the association between submission of BP measurements by the patients during pregnancy and postpartum and by race adjusting for the treatment group (CMOM/usual care), truncated negative binomial models were fitted and expressed as rate ratios and their associated 95% CIs. A risk ratio was used to compare the proportion of Black and White patients diagnosed with a hypertensive disorder of pregnancy who had a blood pressure measurement taken within 7 days of discharge from the hospital. Other confounders (history of diabetes, smoking status, and body mass index [BMI]) identified during descriptive analysis were adjusted for in the models. Statistical analysis was performed using a 5% level of significance (SAS version 9.4, SAS Institute, Inc).

## RESULTS

Using the EHR, 58,216 pregnancies were identified from 47,128 unique, potentially eligible individuals. After eligibility, inclusion, and exclusion criteria were applied, 12,023 patients remained. Patients with a self-reported race other than Black or White, including unknown or missing, were excluded (n=942). A total of 11,081 patients were eligible for inclusion in the study. A total of 3,976 patients received the CMOM intervention. After matching, 7,952 patients were included in the analysis. A flow chart of cohort selection is presented in the Figure.

**Figure.**
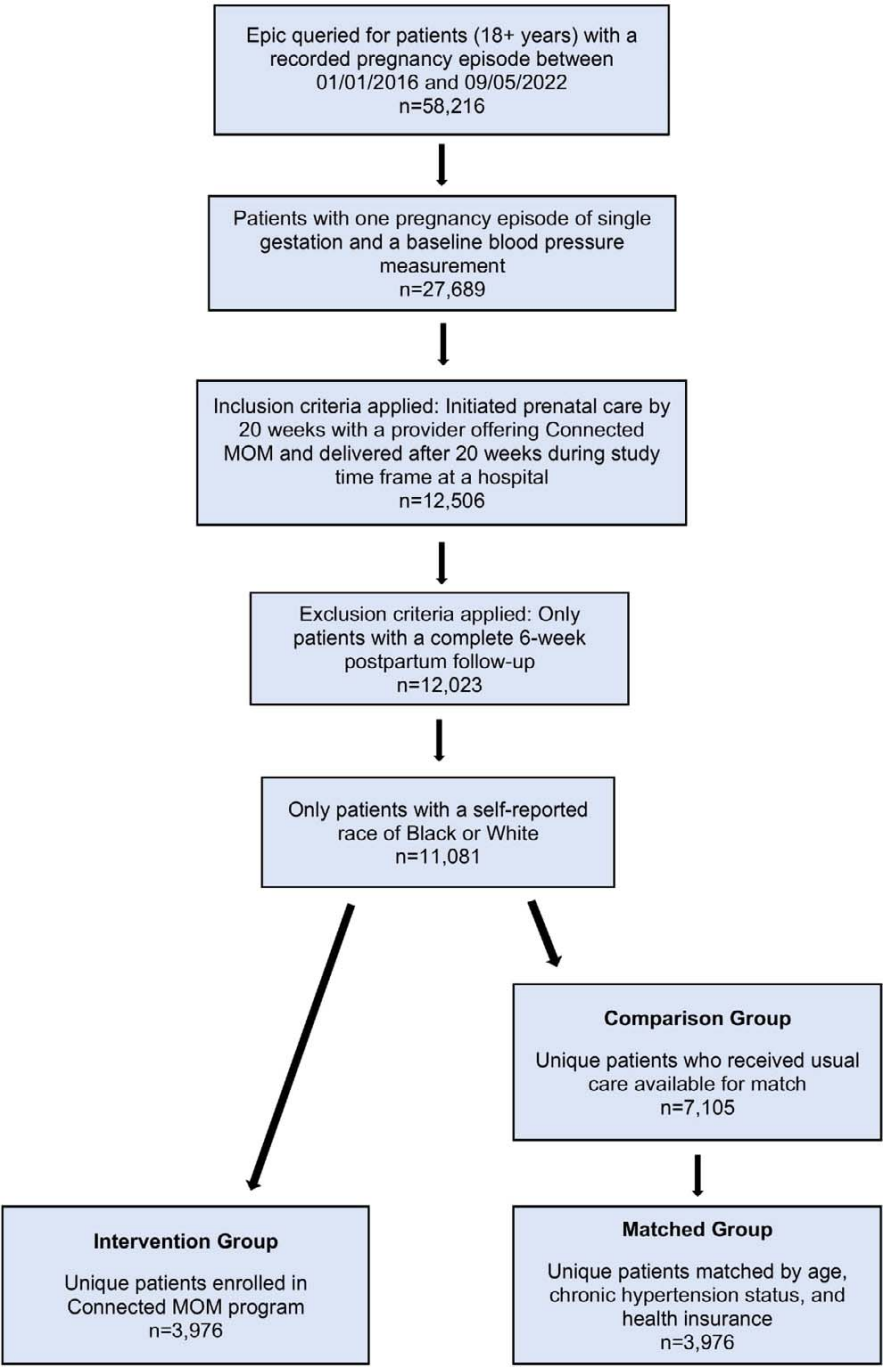
**Flow chart of study participant selection**. MOM, Maternity Online Monitoring.

Demographic and social characteristics of the study patients overall and by treatment group and race are provided in Table 1. Among the cohort (n=7,952), most patients identified as White (62.9%) and non-Hispanic (95.1%). This finding is representative of the obstetrics patient population at this hospital system during the study time period when limiting to only self-identified Black and White races (White 59.8%, non-Hispanic 92.9%). The majority of patients were overweight or obese at or before their initial visit (57.7%, mean BMI of 28.9 ± 7.6 kg/m^2^), nonsmoking (94.1%), and married (62.5%), with an urban residence (98.7%)^26^ and an average age of 30.9 ± 5 years old. Thirteen percent of the cohort used public health insurance (Medicaid or Medicare), with Medicaid accounting for the majority (98.1%).

**Table 1.** Demographic and Social Characteristics of Study Patients, Overall and by Treatment Group and Race

	Overall	CMOM Group				Usual Care Group			
Variable	n=7,952	All, n=3,976	Black, n=1,240	White, n=2,736	*P* Value	All, n=3,976	Black, n=1,708	White, n=2,268	*P* Value
**Demographic**									
Maternal age, years, mean ± SD	30.9 ± 5	30.9 ± 5	29.5 ± 5.5	31.6 ± 4.6	<0.001	30.9 ± 5.1	29.9 ± 5.4	31.7 ± 4.7	<0.001
Body mass index category					<0.001				<0.001
Underweight	722 (9.1)	280 (7.0)	77 (6.2)	203 (7.4)		442 (11.1)	184 (10.8)	258 (11.4)	
Normal	2,552 (32.1)	1,383 (34.8)	262 (21.1)	1,121 (41)		1,169 (29.4)	324 (19.0)	845 (37.3)	
Overweight	1,959 (24.6)	1,032 (26.0)	307 (24.8)	725 (26.5)		927 (23.3)	376 (22.0)	551 (24.3)	
Obese	2,631 (33.1)	1,226 (30.8)	579 (46.7)	647 (23.6)		1,405 (35.3)	812 (47.5)	593 (26.1)	
Ethnicity					<0.001				<0.001
Not Hispanic or Latina	7,566 (95.1)	3,796 (95.5)	1,225 (98.8)	2,571 (94.0)		3,770 (94.8)	1,688 (98.8)	2,082 (91.8)	
Hispanic or Latina	347 (4.4)	159 (4.0)	12 (1.0)	147 (5.4)		188 (4.7)	18 (1.1)	170 (7.5)	
Unknown	39 (0.5)	21 (0.5)	3 (0.2)	18 (0.7)		18 (0.5)	2 (0.1)	16 (0.7)	
**Social**									
Health insurance					<0.001				<0.001
Public^a^	1,031 (13.0)	512 (12.9)	326 (26.3)	186 (6.8)		519 (13.1)	348 (20.4)	171 (7.5)	
Commercial	6,644 (83.6)	3,324 (83.6)	876 (70.6)	2,448 (89.5)		3,320 (83.5)	1,306 (76.5)	2,014 (88.8)	
Other	277 (3.5)	140 (3.5)	38 (3.1)	102 (3.7)		137 (3.4)	54 (3.2)	83 (3.7)	
Smoking status					0.479				0.236
Current smoker	437 (5.5)	171 (4.3)	49 (4.0)	122 (4.5)		266 (6.7)	105 (6.1)	161 (7.1)	
Nonsmoker	7,483 (94.1)	3,793 (95.4)	1,184 (95.5)	2,609 (95.4)		3,690 (92.8)	1,594 (93.3)	2,096 (92.4)	
Marital status					<0.001				<0.001
Never married	2,778 (34.9)	1,180 (29.7)	787 (63.5)	393 (14.4)		1,598 (40.2)	1,173 (68.7)	425 (18.7)	
Divorced, separated, widowed	174 (2.2)	58 (1.5)	15 (1.2)	43 (1.6)		116 (2.9)	55 (3.2)	61 (2.7)	
Married, significant other	4,967 (62.5)	2,726 (68.6)	431 (34.8)	2,295 (83.9)		2,241 (56.4)	467 (27.3)	1,774 (78.2)	
Residence^b^					0.686				0.060
Urban	7,852 (98.7)	3,937 (99.0)	1,229 (99.1)	2,708 (99.0)		3,915 (98.5)	1,689 (98.9)	2,226 (98.1)	
Rural	100 (1.3)	39 (1.0)	11 (0.9)	28 (1.0)		61 (1.5)	19 (1.1)	42 (1.9)	

^a^Public health insurance includes Medicare (1.9%) and Medicaid (98.1%).

^b^The patients’ ZIP codes of residence were used to create the urban-rural variable, as defined by the 2013 Rural-Urban Continuum Codes.^26^

Notes: Data are presented as n (%), unless otherwise indicated. Missing numbers are as follows: body mass index category, n=88; smoking status, n=32; marital status, n=32.

CMOM, Connected Maternity Online Monitoring.

The vast majority (91.5%) had controlled BP at their initial prenatal visit (BP <140/90 mm Hg), and patients achieved an average gestation of 39.1 ± 2.5 weeks (Table 2). Overall, 6.7% of patients had preexisting hypertension, and 28.6% of patients were diagnosed with a hypertensive disorder of pregnancy.

**Table 2.** Maternal Health Characteristics of Study Patients, Overall and by Treatment Group and Race

	Overall	CMOM Group				Usual Care Group			
		All,	Black,	White,		All,	Black,	White,	
Variable	n=7,952	n=3,976	n=1,240	n=2,736	*P* Value	n=3,976	n=1,708	n=2,268	*P* Value
**Baseline health**									
Systolic blood pressure, mm Hg, mean ± SD	117.3 ± 13.3	116.9 ± 13.1	119.5 ± 13.5	115.7 ± 12.8	<0.001	117.7 ± 13.6	121.2 ± 14.5	115.1 ± 12.1	<0.001
Diastolic blood pressure, mm Hg, mean ± SD	72.9 ± 9.2	73.0 ± 9.1	73.5 ± 9.2	72.8 ± 9.1	0.013	72.8 ± 9.3	74.1 ± 9.8	71.9 ± 8.7	<0.001
Blood pressure status at initial PNC visit					0.009				<0.001
Uncontrolled	673 (8.5)	330 (8.3)	124 (10.0)	206 (7.5)		343 (8.6)	212 (12.4)	131 (5.8)	
Controlled	7,279 (91.5)	3,646 (91.7)	1,116 (90.0)	2,530 (92.5)		3,633 (91.4)	1,496 (87.6)	2,137 (94.2)	
Antihypertensive prescription use at initial PNC visit^a^	1,531 (19.3)	738 (18.6)	333 (26.9)	405 (14.8)	<0.001	793 (19.9)	464 (27.2)	329 (14.5)	<0.001
**Maternal health**									
Length of gestation, weeks, mean ± SD	39.1 ± 2.5	39.1 ± 2.4	39.3 ± 2.2	38.8 ± 2.8	<0.001	39.0 ± 2.7	38.6 ± 3.1	39.3 ± 2.2	<0.001
Diabetes									
Preexisting^b^	134 (1.7)	55 (1.4)	28 (2.3)	27 (1.0)	0.002	79 (2.0)	55 (3.2)	24 (1.1)	<0.001
Gestational	539 (6.8)	265 (6.7)	91 (7.3)	174 (6.4)	0.252	274 (6.9)	120 (7.0)	154 (6.8)	0.772
Preexisting hypertension	530 (6.7)	268 (6.7)	132 (10.6)	136 (5.0)	<0.001	262 (6.6)	203 (11.9)	59 (2.6)	<0.001
Preexisting hypertension with preeclampsia	122 (1.5)	66 (1.7)	28 (2.3)	38 (1.4)	0.047	56 (1.4)	48 (2.8)	8 (0.4)	<0.001
Gestational hypertension without proteinuria	1,024 (12.9)	530 (13.3)	170 (13.7)	360 (13.2)	0.635	494 (12.4)	207 (12.1)	287 (12.7)	0.613
Preeclampsia^c^	886 (11.1)	415 (10.4)	163 (13.1)	252 (9.2)	0.001	471 (11.8)	253 (14.8)	218 (9.6)	<0.001
Without severe features	77 (1.0)	33 (0.8)	13 (1.0)	20 (0.7)	0.307	44 (1.1)	18 (1.1)	26 (1.1)	0.783
With severe features	415 (5.2)	208 (5.2)	74 (6.0)	134 (4.9)	0.160	207 (5.2)	114 (6.7)	93 (4.1)	<0.001
HELLP syndrome	24 (0.3)	9 (0.2)	3 (0.2)	6 (0.2)	>0.999	15 (0.4)	5 (0.3)	10 (0.4)	0.604
Eclampsia	12 (0.2)	5 (0.1)	4 (0.3)	1 (0)	0.035	7 (0.2)	3 (0.2)	4 (0.2)	>0.999
Pregnancy-associated hypertension^d^	1,647 (20.7)	822 (20.7)	293 (23.6)	529 (19.3)	0.002	825 (20.7)	402 (23.5)	423 (18.7)	0.001
Hypertensive disorder of pregnancy^e^	2,276 (28.6)	1,141 (28.7)	421 (34.0)	720 (26.3)	<0.001	1,135 (28.5)	606 (35.5)	529 (23.3)	<0.001
In-hospital mortality					NA				0.581
Deceased	4 (0.1)	1 (<0.1)	0 (0)	1 (<0.1)		3 (0.1)	2 (0.1)	1 (<0.1)	
Alive	7,948 (99.9)	3,975 (>99.9)	1,240 (100)	2,735 (>99.9)		3,973 (99.9)	1,706 (99.9)	2,267 (>99.9)	

^a^Antihypertensive medications included labetalol, amlodipine, methyldopa, and nifedipine.

^b^Preexisting diabetes includes type 1, type 2, or unspecified diabetes mellitus in pregnancy, childbirth, and the puerperium.

^c^The subcategories of preeclampsia do not sum because an additional category, unspecified preeclampsia, is not presented in the table.

^d^Pregnancy-associated hypertension includes gestational hypertension without proteinuria, preeclampsia, eclampsia, and preexisting hypertension with superimposed preeclampsia. These components do not sum to the total shown for pregnancy-associated hypertension because an individual patient may have >1 diagnosis/condition.

^e^Hypertensive disorder of pregnancy includes preexisting hypertension, pregnancy-associated hypertension, or unspecified maternal hypertension (not included in the table).

Note: Data are presented as n (%), unless otherwise indicated.

CMOM, Connected Maternity Online Monitoring; HELLP, hemolysis, elevated liver enzymes, and low platelet count; NA, not applicable; PNC, prenatal care.

In the cohort, Black patients were younger (29.7 vs 31.6 years old, *P*<0.0001) and more likely to be never married (66.5% vs 16.3%, *P*<0.0001), obese (47.2% vs 24.8%, *P*<0.0001), and use public insurance (22.9% vs 7.1%, *P*<0.0001) than their White counterparts. Black patients were less likely to be enrolled in CMOM than White patients (42.1% vs 54.7%, *P*<0.0001). This finding was also seen in the eligible cohort before matching (n=11,081): 26.9% of Black patients enrolled in CMOM vs 42.3% of White patients (*P*<0.0001).

CMOM participants compared to the usual care group were more likely to be married (68.6% vs 56.4%, *P*<0.0001) and have a normal BMI (34.8% vs 29.4%, *P*<0.0001), and they were less likely to smoke (4.3% vs 6.7%, *P*<0.0001). The usual care group had more patients with preexisting diabetes (2.0% vs 1.4%, *P*=0.0365) and more diagnoses of preeclampsia (11.8% vs 10.4%, *P*=0.046) than the CMOM group. Length of gestation, baseline BP, and rates of hypertensive disorder of pregnancy were similar between the groups.

Black patients in CMOM were more likely to have BMIs in the normal range (21.1% vs 19.0%, *P*=0.0001), less likely to smoke (4.0% vs 6.1%, *P*=0.0082), to have preexisting hypertension (10.6% vs 11.9%, *P*=0.2950), and to have uncontrolled BP at baseline (10.0% vs 12.4%, *P*=0.0419) than Black patients in usual care. However, compared to White patients in CMOM, Black patients in CMOM had higher rates of obesity (46.7% vs 23.6%, *P*<0.0001), preexisting hypertension (10.6% vs 5.0%, *P*<0.0001), uncontrolled BP (10.0% vs 7.5%, *P*=0.0089), and diabetes (2.3% vs 1.0%, *P*=0.002). White patients with preexisting hypertension and uncontrolled hypertension at baseline were more likely to be enrolled in CMOM than usual care (5.0% vs 2.6%, *P*<0.0001; 7.5% vs 5.8%, *P*=0.0138, respectively), whereas Black patients with those diagnoses were less likely to be enrolled in CMOM than usual care (10.6% vs 11.9%, *P*=0.2950; 10.0% vs 12.4%, *P*=0.0419, respectively).

Patients in the CMOM group, irrespective of race, had 1.8 times more BP measurements than usual care patients during pregnancy (26 vs 15 measurements; rate ratio=1.78, 95% CI 1.74-1.82). Patients enrolled in CMOM had shorter intervals between BP measurements compared to usual care patients (12 vs 17 days, *P*<0.001) (Table 3). Black patients enrolled in CMOM measured their BP more frequently (23 vs 15 measurements, *P*<0.001) and had a shorter interval between measurements (13 days vs 17 days, *P*<0.001) than Black patients in the usual care group. However, compared to White patients enrolled in CMOM, Black patients enrolled in CMOM submitted fewer BP measurements (23 vs 28 measurements, *P*<0.001) and less often (every 13 vs 11 days, *P*<0.001).

**Table 3.** Blood Pressure Ascertainment and Intervals During Pregnancy and Postpartum, Overall and by Treatment Group and Race^a^

	All Patients		Black Patients			White Patients		
	CMOM	Usual Care	CMOM	Usual Care		CMOM	Usual Care	
Time Period/Variable	Group	Group	Group	Group	*P* Value	Group	Group	*P* Value
**During pregnancy**	**n=3,976**	**n=3,976**	**n=1,240**	**n=1,708**		**n=2,736**	**n=2,268**	
Blood pressure ascertainment: Number of measurements	26 ± 16	15 ± 5	23 ± 13	15 ± 6	<0.001	28 ± 17	14 ± 5	<0.001
Blood pressure interval: Time interval between measurements, days	12 ± 6	17 ± 6	13 ± 6	17 ± 6	<0.001	11 ± 6	18 ± 6	<0.001
**During 6-week postpartum period^b^**	**n=1,108**	**n=1,112**	**n=404**	**n=597**		**n=704**	**n=515**	
Blood pressure ascertainment: Number of measurements	6 ± 5	5 ± 2	6 ± 4	5 ± 3	0.001	6 ± 5	5 ± 2	<0.001
Blood pressure interval: Time interval between measurements, days	7 ± 6	7 ± 6	6 ± 5	7 ± 6	0.061	7 ± 7	8 ± 7	0.002

^a^For blood pressure ascertainment and blood pressure interval, the Wilcoxon rank sum test was used.

^b^Only patients with a diagnosed hypertensive disorder of pregnancy during the current pregnancy (*International Classification of Diseases-10* diagnosis codes O10, O11, O13, O14, O15, O16) and 2 or more blood pressure measurements in the postpartum period were included to allow for interval calculation. Six weeks following delivery was chosen to reflect the traditional comprehensive postpartum visit.

Note: Data are presented as mean ± SD.

CMOM, Connected Maternity Online Monitoring.

During the postpartum period, patients in the CMOM group who had been diagnosed with hypertensive disorders of pregnancy, irrespective of race, had more BP measurements than usual care patients (6 vs 5 measurements; rate ratio=1.30, 95% CI 1.23-1.37), with statistically significant differences seen among both Black and White patients (Table 3). Patients in CMOM and usual care had similar intervals (7 days) between BP measurements during the 6-week postpartum period. Although not statistically significant, Black patients with hypertensive disorders of pregnancy in CMOM had better adherence to BP ascertainment than White patients in CMOM, taking their BP every 6 days vs 7 days during the postpartum period (*P*=0.98).

Patients with a diagnosis of hypertensive disorder of pregnancy who enrolled in CMOM were modestly more likely to take their BP within 7 days of discharge from the hospital than usual care patients (71.6% vs 67.6%, respectively, risk ratio=1.06, 95% CI 1.00-1.11); however, the CMOM intervention did not result in an improvement in 7-day postpartum adherence for Black patients (risk ratio=1.03, 95% CI 0.94-1.11) as it did for White patients (risk ratio=1.09, 95% CI 1.01-1.17) (Table 4).

**Table 4.** Proportion of Patients Diagnosed With a Hypertensive Disorder of Pregnancy With a Blood Pressure Measurement Taken Within 7 Days of Discharge From the Hospital, n=2,276

	Blood Pressure Measurement Within 7 Days of Delivery Discharge				
Patient Group	Total	Yes	No	Risk Ratio (95% CI)^a^	*P* Value
Overall					0.0368
CMOM group	1,141	817 (71.6)	324 (28.4)	1.06 (1.00-1.11)	
Usual care group	1,135	767 (67.6)	368 (32.4)		
Total	2,276	1,584 (69.6)	692 (30.4)		
Black patients					0.550
CMOM group	421	295 (70.1)	126 (29.9)	1.03 (0.94-1.11)	
Usual care group	606	414 (68.3)	192 (31.7)		
Total	1,027	709 (69.0)	318 (31.0)		
White patients					0.0278
CMOM group	720	522 (72.5)	198 (27.5)	1.09 (1.01-1.17)	
Usual care group	529	353 (66.7)	176 (33.3)		
Total	1,249	875 (70.0)	374 (29.9)		

^a^Chi-square test analysis.

Notes: Data are presented as n (%). Percentages are calculated across rows.

CMOM, Connected Maternity Online Monitoring.

## DISCUSSION

In this study, we demonstrate the effectiveness of a remote BP monitoring program to frequently monitor BP during pregnancy and postpartum, while also highlighting the importance of equitable implementation. Regardless of race, CMOM program participants documented their BP more often and more routinely than patients in the usual care group during both pregnancy and in the postpartum period. Black patients enrolled in CMOM had more frequent BP ascertainment and shorter intervals between measurements during pregnancy and in the postpartum period compared to Black patients in usual care. Although not statistically significant for Black patients, patients in the CMOM group with a hypertensive disorder of pregnancy were more likely to take their BP within 7 days of discharge from the hospital than patients in the usual care group.

For Black patients, the ability to improve BP monitoring, especially in the postpartum period, is important for reducing maternal morbidity and mortality. In a review of the National Inpatient Sample (as of 2014), the rate of preeclampsia among Black patients was 69.8 per 1,000 deliveries vs 43.3 per 1,000 deliveries among White patients.^27^ In a study measuring racial differences in postpartum BP among individuals with hypertensive disorders of pregnancy, Black patients were found to have a slower return to normotensive BP during the postpartum period,^28^ highlighting the importance of BP monitoring for Black patients to reduce maternal morbidity and mortality. Changing BP cutoffs and definitions over time and by country have made reaching a consensus for managing hypertensive disorders of pregnancy during the prenatal and postpartum periods difficult. In 2022, the American College of Obstetricians and Gynecologists, through the Alliance for Innovation on Maternal Health, updated their recommendation for postpartum BP evaluation.^25,29^ According to the recommendation, patients with severe hypertension, defined as systolic BP >160 mm Hg and/or diastolic BP >100 mm Hg, should have a follow-up BP measurement 3 days after discharge from the hospital. This shortened time frame may be difficult to accomplish given the barriers to accessing care in the postpartum period.^30^ Remote BP monitoring has the potential to improve compliance with this recommendation and to potentially identify patients who have a higher lifetime risk of cardiovascular morbidity and mortality.

The effectiveness of remote monitoring to improve adherence to BP evaluation has been demonstrated in the literature.^24,28,31^ However, unlike previous studies, we specifically evaluated engagement among Black patients. While CMOM has the potential to improve outcomes, we did not see equivalent enrollment. Black patients, regardless of health status, were less likely to enroll in CMOM compared to their White counterparts. There may be a few explanations for this disparity, which will require future research. Participation in the program was at the discretion of the provider to offer enrollment, which may have impacted offer rates. Implicit bias has been shown to impact health care delivery in offering preventive services, as well as provider decision-making.^32-34^ More research is needed to determine if CMOM is offered at equitable rates for Black patients. Health care providers should use standardized, patient-centered scripting to remove bias in how patients are offered enrollment.

Understanding the role of the digital divide in this population is imperative. Participation in CMOM requires a smartphone and an internet connection. Americans with low incomes (<$30,000) have lower levels of smartphone ownership (76% vs 97%) and access to home broadband (57% vs 93%) than Americans with high incomes (>$100,000),^35^ and Black adults are less likely than White adults to have home broadband internet (71% vs 80%).^36^ Future evaluation work on CMOM must assess the role of access to smartphones, data plans, and the internet on enrollment in the program and whether there is a differential by race and income.

All patients who enroll in CMOM are offered technical assistance, but technical assistance does not address digital health literacy which is often a barrier to engagement. Research has documented decreased engagement with telehealth and wearable devices among Black patients compared to White patients,^37,38^ but engagement can be improved with increased training on digital devices.^39^ When patients decline participation or do not complete the onboarding phase, follow-up questions are needed to determine if training on the devices or addressing other digital barriers can improve enrollment.

This study has several limitations. Patients were not prospectively randomized into the CMOM and usual care groups. The study was limited to previously collected health data from the EHR which may have documentation inaccuracies, coding errors, or missing information; however, extensive data quality tests were performed, and missing data were minimal. Only patients who possessed a smartphone were eligible to enroll in the program, which could have created a selection bias. To minimize bias, the groups were matched by health insurance as a proxy for income. A larger proportion of patients in this dataset had commercial insurance compared to the pregnant population in the United States, which limits the generalizability of our findings. This analysis did not adjust for the effects of current events (eg, the coronavirus disease 2019 pandemic, hurricanes affecting the Gulf South).

Assessing BP control during pregnancy, timeliness of office visits following abnormal BP measurements, and associated adverse maternal outcomes were beyond the scope of this process of care analysis. BP control is not a CMOM program goal. The program relies on remote measurements (process outcomes) to inform clinical decision-making that may or may not result in detectable BP changes in EHR data. While CMOM has the potential to improve outcomes through frequent monitoring of changing health, determining the effect of CMOM on distal adverse maternal health outcomes is better suited for a prospective study design.

## CONCLUSION

The remote monitoring program, CMOM, is a helpful tool to improve BP ascertainment and shorten intervals between measurements during the prenatal and postpartum periods for all patients. By frequently monitoring their BP, patients in CMOM provided additional data to their obstetrics provider for clinical decision-making. This research found that the program is beneficial for Black patients and improves, but does not completely eliminate, racial disparities in BP ascertainment. Digital technology presents an innovative way to bring care to patients, but we must examine this care model through a lens of equity to ensure all individuals have access and are given the ability to participate. Future research plans include analysis of prenatal and postpartum BP control and adverse maternal outcomes, the long-term benefits of program participation, and the correlation with enrollment in other digital medicine programs for patients with preexisting hypertension. Finally, research on equitable implementation strategies is necessary to optimize enrollment and utilization of remote BP monitoring programs such as CMOM in high-risk populations.

## Notes

*This article meets the Accreditation Council for Graduate Medical Education and the American Board of Medical Specialties Maintenance of Certification competencies for Patient Care, Medical Knowledge, Systems-Based Practice, and Practice-Based Learning and Improvement.*
